# A nomogram for predicting survival in patients with de novo metastatic breast cancer: a population-based study

**DOI:** 10.1186/s12885-020-07449-1

**Published:** 2020-10-12

**Authors:** Wen Zhao, Lei Wu, Andi Zhao, Mi Zhang, Qi Tian, Yanwei Shen, Fan Wang, Biyuan Wang, Le Wang, Ling Chen, Xiaoai Zhao, Danfeng Dong, Lingxiao Zhang, Jin Yang

**Affiliations:** Department of Oncology, the First Affiliated Hospital of Xi’an Jiaotong University, No 277 Yenta West Road, Xi’an, Shaanxi 710061 People’s Republic of China

**Keywords:** De novo metastatic breast cancer, Primary tumor resection, Overall survival, SEER, Nomogram

## Abstract

**Background:**

5–10% of patients are diagnosed with metastatic breast cancer (MBC) at the initial diagnosis. This study aimed to develop a nomogram to predict the overall survival (OS) of these patients.

**Methods:**

de novo MBC patients diagnosed in 2010–2016 were identified from the Surveillance, Epidemiology, and End Results (SEER) database. They were randomly divided into a training and a validation cohort with a ratio of 2:1. The best subsets of covariates were identified to develop a nomogram predicting OS based on the smallest Akaike Information Criterion (AIC) value in the multivariate Cox models. The discrimination and calibration of the nomogram were evaluated using the Concordance index, the area under the time-dependent receiver operating characteristic curve (AUC) and calibration curves.

**Results:**

In this study, we included 7986 patients with de novo MBC. The median follow-up time was 36 months (range: 0–83 months). Five thousand three-hundred twenty four patients were allocated into the training cohort while 2662 were allocated into the validation cohort. In the training cohort, age at diagnosis, race, marital status, differentiation grade, subtype, T stage, bone metastasis, brain metastasis, liver metastasis, lung metastasis, surgery and chemotherapy were selected to create the nomogram estimating the 1-, 3- and 5- year OS based on the smallest AIC value in the multivariate Cox models. The nomogram achieved a Concordance index of 0.723 (95% CI, 0.713–0.733) in the training cohort and 0.719 (95% CI, 0.705–0.734) in the validation cohort. AUC values of the nomogram indicated good specificity and sensitivity in the training and validation cohort. Calibration curves showed a favorable consistency between the predicted and actual survival probabilities.

**Conclusion:**

The developed nomogram reliably predicted OS in patients with de novo MBC and presented a favorable discrimination ability. While further validation is needed, this may be a useful tool in clinical practice.

## Background

Breast cancer is the most common kind of malignancy in females worldwide; it ranks second in contributing to tumor related death in women [1, 2]. Approximately 266,120 new cases of invasive breast cancer and 40,920 breast cancer deaths were expected to occur among US women in 2018 [1]. 5–10% of patients were diagnosed with metastatic breast cancer (MBC) at the initial diagnosis. Accurately estimating the prognosis of these patients helps greatly in clinical decision-making. However, most prognosis models were developed for early-stage breast cancer [3, 4]. Thus, effective prediction models for de novo MBC patients are warranted to be developed.

Breast cancer tends to be heterogeneous, characterized by diverse histopathologic and molecular features, including age at diagnosis, race, differentiation grade, molecular subtypes, and site of metastasis. These characteristics were previously reported to be associated with survival of de novo MBC patients [5, 6]. Chemotherapy and radiation therapy remain the mainstay for MBC patients. Primary tumor resection is not routinely recommended because MBC is considered an incurable disease [7, 8]; it is only considered as a means of palliation. However, many retrospective analyses reported the survival benefit of primary tumor resection [9–12]. These factors mentioned above may interact, leading to distinct outcomes across individual patients.

A nomogram is a reliable and accurate visualization model utilizing risk factors identified in multivariate analysis; it is widely used for the prediction of survival in oncology [13, 14]. In this study, we developed and validated a nomogram to predict the survival of de novo MBC patients, through a large cohort of well-characterized patients identified from the Surveillance, Epidemiology and End Results (SEER) database.

## Methods

### Patients

Data was obtained from the National Cancer Institute’s Surveillance, Epidemiology, and End Results (SEER) program, which consists of 18 population-based cancer registries, for patients diagnosed between 2010 and 2016. SEER is an open-access resource for tumor-based demographic and pathological information, as well as treatment information and patient survival outcomes. SEER*Stat Version 8.3.4 (http://www.seer.cancer.gov/seerstat) was used to identify eligible patients.

Because the SEER database began collecting information on the human epidermal growth factor receptor-2 (HER2) status and sites of distant metastasis in 2010, this was used as the starting point. The inclusion criteria of MBC patients were listed as follows: female, year of diagnosis from 2010 to 2016, older than 18 years old when diagnosed, breast cancer as the first and only malignant tumor diagnosis, histology of infiltrating duct or/and lobular carcinoma(IDC, ILC), at least one distant site of de novo metastasis. Patients with unknown condition of marital status, race, differentiation grade, T stage, N stage, site of metastasis, or follow-up information were excluded.

Demographic variables including age at diagnosis (<=40, 40–60, and > 60 years), race (white, black, and others, including American Indian/Alaska Native, Asian or Pacific Islander) and marital status (married and unmarried, including divorced, separated, widowed, single (never married) or domestic partner). Tumor characteristics included histology (IDC, ILC, and IDC and ILC), grade (grade I, grade II and grade III/IV), molecular subtype, T stage (T1, T2, T3, and T4), N stage (N0, N1, N2 and N3), and metastatic site (the bone, brain, liver, and lung). Therapies included chemotherapy (No/Unknown and Yes), radiation (No/Unknown and Yes), and surgery of the primary tumor (No, Mastectomy, and Breast conservation surgery (BCS)). Estrogen receptor (ER) and progesterone receptor (PR) status were combined as the hormone receptor (HR) status, and the breast cancer molecular subtype was stratified based on joint HR and human epidermal growth factor receptor-2 (HER2) statuses (HER2−/HR+, HER2+/HR-, HER2+/HR+, and HER2−/HR-).

### Statistical analysis

Patient demographics, tumor characteristics and treatment information were compared using the chi-square test. Overall survival (OS) was defined as the time from breast cancer diagnosis to death from any cause. Patients in the initial cohort were allocated randomly into a training cohort and a validation cohort with a ratio of 2:1. The training cohort was used to develop a nomogram while the validation model was used to validate the model. In the training cohort, the covariates included in the multivariate Cox proportional hazards models were identified by a backward stepwise method based on the smallest Akaike information criterion (AIC) value, which indicated the minimal loss of prognostic information [15, 16].

The nomogram was developed on the basis of independent risk factors and using the “rms” R package. The predictive capacity of the nomogram was assessed using Harrell’s C-index (the concordance statistic, or C-statistic) and the area under the time-dependent receiver operating characteristic curve (AUC), which estimates the probability between the observed and predicted OS. Bootstrapping method with 1000 resamples was utilized to generate the calibration curves for validation of the nomogram in the training cohort and in the validation cohort. The scores of each variable were calculated using the “nomogramEx” package in R. On the basis of the scores of each variable, the total scores for each patient could be calculated.

All analyses were performed with SPSS (version 24.0; SPSS, Inc., Chicago, IL) and R version 3.6.0 (http://www.r-project.org). Statistical significance was assumed at a two-side *p* value of < 0.05.

## Results

### Patient characteristics

**Fig. 1:**
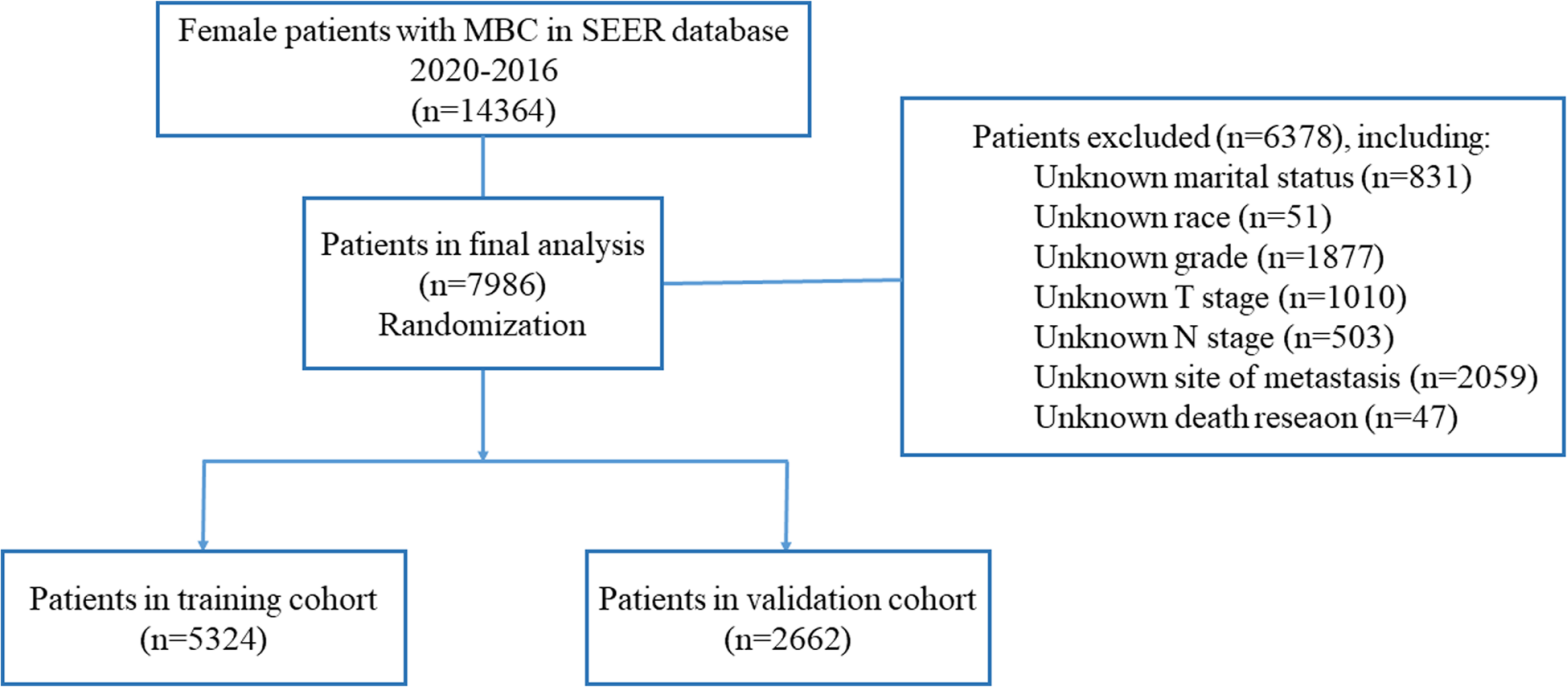
The flowchart of patient selection process

We included 7986 patients with de novo MBC in the final analysis. The flowchart of the patient selection process is shown in Fig. 1. The median follow-up time was 36 months (range: 0–83 months). The median age at diagnosis was 59 years. Most of the patients (75.1%, 5999) were white. 50.3% (4018) of tumors were poorly differentiated or undifferentiated. HR+/HER2- was the most common (57.6%) subtype among MBC patients, followed by HR+/HER2+ (19.0%) and TNBC (triple negative breast cancer) (13.6%) while HR−/HER2+ was the least common (9.8%) subtype. The most common site of metastasis was bone, making up 73.4% (5865) while the least common site of metastasis was brain, making up 6.9% (548). 27.5% (2196) of patients received surgery for the primary tumor, of which 37.9% (832) underwent BCS. 35.0% (2797) of patients received radiotherapy and 61.8% (4937) of patients received chemotherapy. The 1-, 3-, and 5-year OS rates were 74.5, 45.3, and 28.2%, respectively.

The included 7986 patients were allocated randomly into the training cohort (*N* = 5324) and the validation cohort (*N* = 2662). The demographic, pathological and treatment information of the two cohorts is listed in Table 1. The distribution of these factors was balanced in the training and validation cohorts. The median OS of the training and validation cohorts was 38 months (interquartile range, 13–66 months) and 39 months (interquartile range, 12–68 months), respectively.

**Table 1:** The demographic, pathological and treatment information of MBC patients diagnosed at 2010–2016 in the SEER database

Characteristics	The initial cohort		The training cohort		The validating cohort		*p* value
	Number	Percent (%)	Number	Percent (%)	Number	Percent (%)	
Age							0.873
<=40	824	10.3	556	10.4	268	10.1	
<=60	3462	43.4	2305	43.3	1157	43.5	
> 60	3700	46.3	2463	46.3	1237	46.5	
Race							0.160
White	5999	75.1	4016	75.4	1983	74.5	
Black	1339	16.8	898	16.9	441	16.6	
Others^a^	648	8.1	410	7.7	238	8.9	
Marital status							0.837
Unmarried^b^	4208	52.7	2801	52.6	1407	52.9	
Married	3778	47.3	2523	47.4	1255	47.1	
Histology							0.615
IDC	6845	85.7	4571	85.9	2274	85.4	
ILC	737	9.2	480	9.0	257	9.7	
IDC and ILC	404	5.1	273	5.1	131	4.9	
Grade							0.936
I	571	7.2	377	7.1	194	7.3	
II	3397	42.5	2269	42.6	1128	42.4	
III/IV	4018	50.3	2678	50.3	1340	50.3	
Subtype							0.431
HR+/HER2-	4602	57.6	3073	57.7	1529	57.4	
HR−/HER2+	782	9.8	504	9.5	278	10.4	
HR+/HER2+	1518	19.0	1009	19.0	509	19.1	
HR−/HER2-	1084	13.6	738	13.9	346	13.0	
T stage							0.524
T1	922	11.5	616	11.6	306	11.5	
T2	2829	35.4	1857	34.9	972	36.5	
T3	1514	19.0	1015	19.1	499	18.7	
T4	2721	34.1	1836	34.5	885	33.2	
N stage							0.335
N0	1740	21.8	1159	21.8	581	21.8	
N1	3856	48.3	2538	47.7	1318	49.5	
N2	1093	13.7	743	14.0	350	13.1	
N3	1297	16.2	884	16.6	413	15.5	
Bone metastasis							0.420
no	2121	26.6	1399	26.3	722	27.1	
yes	5865	73.4	3925	73.7	1940	72.9	
Brain metastasis							0.661
no	7438	93.1	4954	93.1	2484	93.3	
yes	548	6.9	370	6.9	178	6.7	
Liver metastasis							0.716
no	5616	70.3	3737	70.2	1879	70.6	
yes	2370	29.7	1587	29.8	783	29.4	
Lung metastasis							0.907
no	5231	65.5	3485	65.5	1746	65.6	
yes	2755	34.5	1839	34.5	916	34.4	
Surgery							0.661
No	5790	72.5	3843	72.2	1947	73.1	
BCS	832	10.4	560	10.5	272	10.2	
Mastectomy	1364	17.1	921	17.3	443	16.6	
Radiotherapy							0.445
No/Unknown	5189	65.0	3444	64.7	1745	65.6	
Yes	2797	35.0	1880	35.3	917	34.4	
Chemotherapy							0.948
No/Unknown	3049	38.2	2034	38.2	1015	38.1	
Yes	4937	61.8	3290	61.8	1647	61.9	

^a^Other races included American Indian/Alaska Native, Asian or Pacific Islander

^b^Unmarried included divorced, separated, widowed, single (never married) or domestic partner

### Nomogram construction

**Fig. 2:**
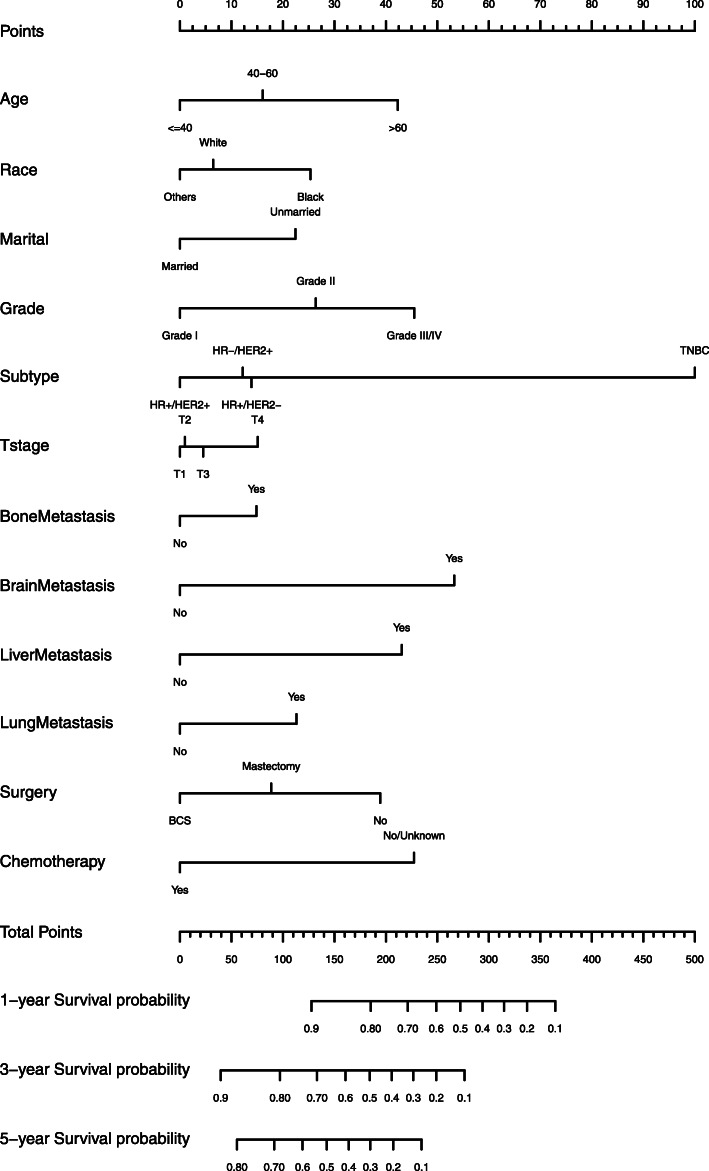
Nomogram predicted 1-, 3- and 5-year overall survival for de novo MBC patients

According to univariate analysis, age at diagnosis, race, marital status, differentiation grade, molecular subtype, T stage, bone metastasis, brain metastasis, liver metastasis, lung metastasis, surgery, radiotherapy and chemotherapy were associated with OS (*p* < 0.05, Table 2). The smallest AIC value occurred when we incorporated 12 factors into the multivariate Cox analysis: age at diagnosis, race, marital status, differentiation grade, molecular subtype, T stage, bone metastasis, brain metastasis, liver metastasis, lung metastasis, surgery and chemotherapy (AIC = 6606.9). Figure 2 shows the prediction of the 1-, 3- and 5-year OS probability in the nomogram. Every specific value of these factors was allocated a score on the points scale. By adding up these scores, the total score was calculated. The total points was used to estimate the 1-, 3- and 5-year survival probability for every individual patient.

**Table 2:** The prognostic factors identified in the univariate and multivariate Cox regression models in the training cohort incorporating covariates identified by the smallest AIC value

Characteristics	Univariate Cox analysis				Multivariate Cox analysis			
	Hazard ratio	95%CI		*p* value	Hazard ratio	95%CI		*p* value
Age								
< =40	R			< 0.001	R			< 0.001
< =60	1.328	1.148	1.537	< 0.001	1.231	1.062	1.427	0.006
> 60	1.870	1.621	2.159	< 0.001	1.724	1.483	2.003	< 0.001
Race								
White	R			< 0.001	R			< 0.001
black	1.394	1.268	1.533	< 0.001	1.275	1.154	1.407	< 0.001
Others^a^	0.884	0.756	1.033	0.122	0.922	0.788	1.079	0.311
Marital status								
Unmarried^b^	R			< 0.001	R			< 0.001
Married	0.666	0.616	0.719		0.751	0.694	0.813	
Histology								
IDC	R			0.189	Not included			
ILC	0.916	0.801	1.048	0.202				
IDC and ILC	0.882	0.738	1.053	0.164				
Grade								
I	R			< 0.001	R			< 0.001
II	1.228	1.036	1.454	0.018	1.401	1.180	1.662	< 0.001
III/IV	1.743	1.476	2.058	< 0.001	1.792	1.504	2.135	< 0.001
Subtype								
HR+/HER2-	R			< 0.001	R			< 0.001
HR−/HER2+	0.988	0.860	1.135	0.862	0.972	0.835	1.132	0.715
HR+/HER2+	0.801	0.717	0.895	< 0.001	0.832	0.739	0.938	0.003
HR−/HER2-	1.926	2.649	3.232	< 0.001	2.969	2.642	3.337	< 0.001
T stage								
T1	R			< 0.001	R			0.001
T2	1.005	0.878	1.151	0.938	1.011	0.882	1.159	0.875
T3	1.162	1.003	1.345	0.045	1.059	0.913	1.229	0.450
T4	1.507	1.321	1.720	< 0.001	1.213	1.059	1.389	0.005
N stage								
N0	R			0.244	Not included			
N1	0.930	0.811	1.066	0.298				
N2	0.827	0.685	0.998	0.047				
N3	0.966	0.810	1.151	0.696				
Bone metastasis								
No	R			< 0.001	R			< 0.001
Yes	0.856	0.787	0.932		1.208	1.099	1.327	
Brain metastasis								
No	R			< 0.001	R			< 0.001
Yes	2.314	2.040	2.626		1.962	1.724	2.233	
Liver metastasis								
No	R			< 0.001	R			< 0.001
Yes	1.534	1.416	1.661		1.728	1.586	1.883	
Lung metastasis								
No	R			< 0.001	R			< 0.001
Yes	1.570	1.453	1.697		1.335	1.228	1.452	
Surgery								
No	R			< 0.001	R			< 0.001
BCS	0.547	0.476	0.628	< 0.001	0.606	0.526	0.699	< 0.001
Mastectomy	0.724	0.654	0.802	< 0.001	0.762	0.686	0.845	< 0.001
Radiotherapy								
No/Unknown	R			0.003	Not included			
Yes	0.885	0.818	0.958					
chemotherapy								
No/Unknown	R			< 0.001	R			< 0.001
Yes	0.674	0.624	0.727		0.564	0.516	0.617	

^a^Other races included American Indian/Alaska Native, Asian or Pacific Islander

^b^Unmarried included divorced, separated, widowed, single (never married) or domestic partner

### Nomogram validation and calibration

**Fig. 3:**
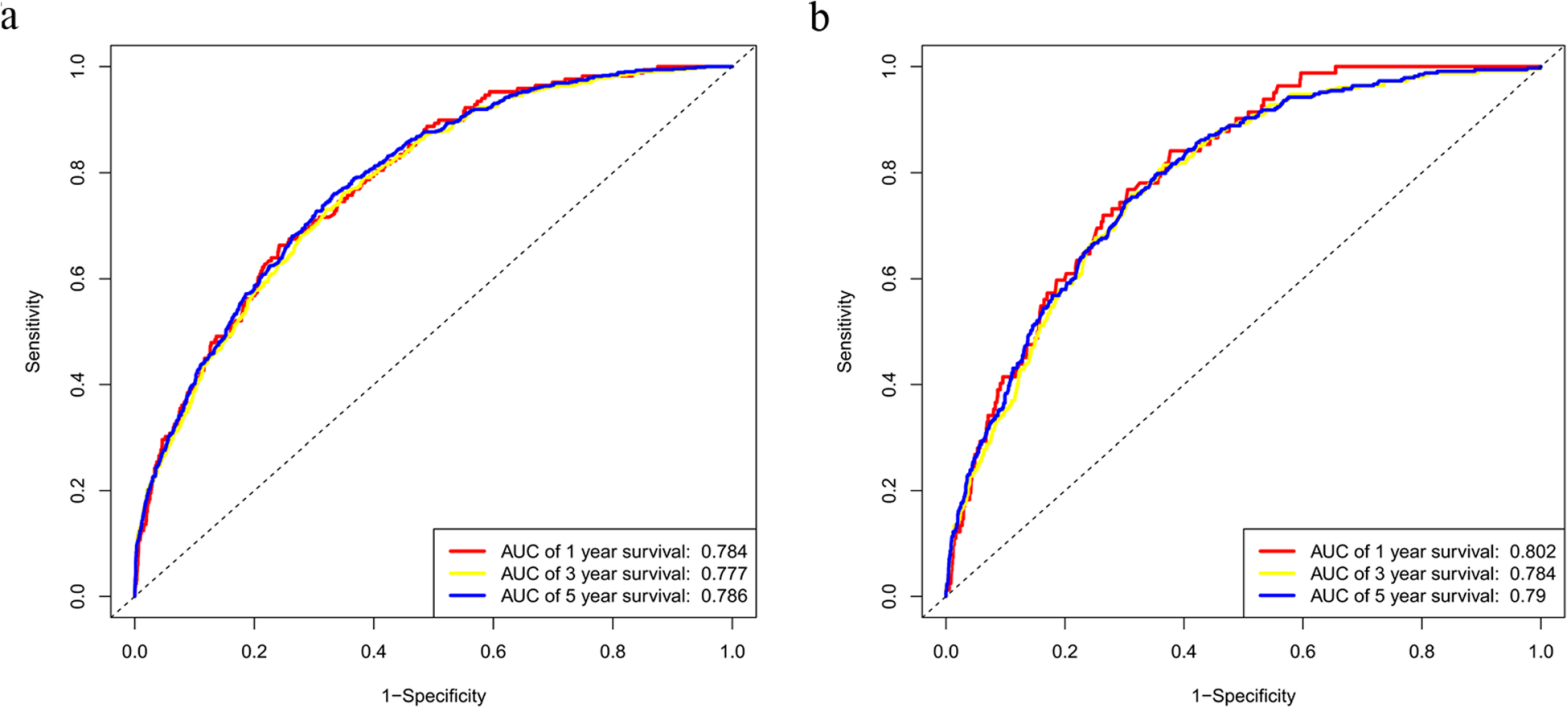
1-, 3 -, and 5-years receiver operating characteristic curves in training **a** and validation cohorts **b**

**Fig. 4:**
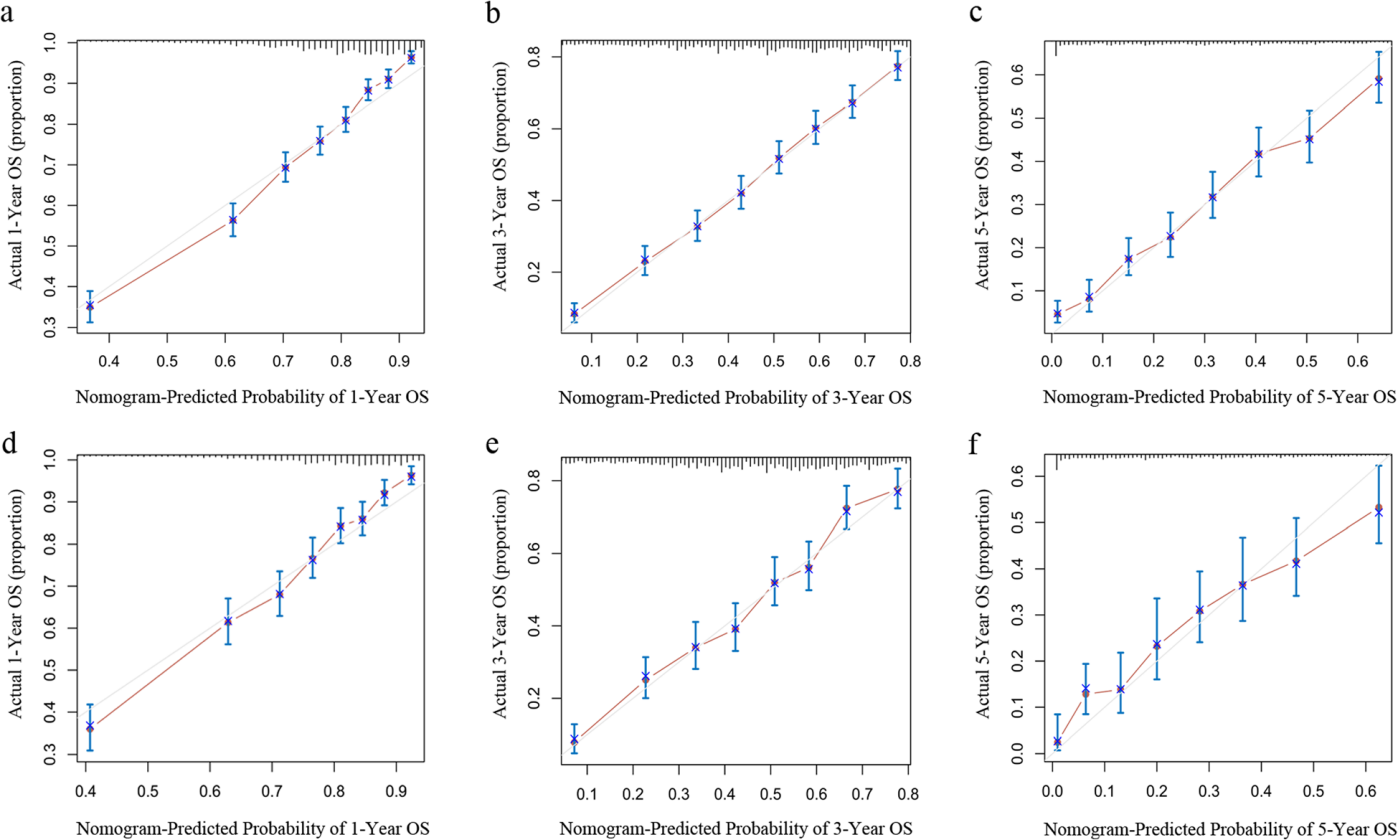
The calibration plots for predicting patient survival at 1-, 3- and 5-year point in the training cohort **a**, **b**, **c**) and the validation cohort (**d**, **e**, **f**)

The nomogram was validated in the training cohort and in the validation cohort, respectively. The C-index was 0.723 (95% CI, 0.713–0.733) and 0.719 (95% CI, 0.705–0.734) in the training and validation cohort, respectively. In the training cohort, the AUC values of the nomogram to predict 1-, 3- and 5-year OS was 0.784 (95% CI, 0.752–0.816), 0.777 (95% CI, 0.757–0.798) and 0.786 (95% CI, 0.768–0.803), respectively. In the validation cohort, the AUC values of the nomogram to predict 1-, 3- and 5-year OS was 0.802 (95% CI, 0.762–0.841), 0.784 (95% CI, 0.757–0.811) and 0.790 (95% CI, 0.765–0.814), respectively (Fig. 3). The calibration plots for the probability of OS indicated an optimal agreement between 1-, 3- and 5-year prediction by nomogram and observation in both training cohort and validation set (Fig. 4).

## Discussion

The survival of patients with de novo MBC is difficult to predict, because of the lack of prediction models for these patients. In this study, we developed a nomogram to visualize survival of de novo MBC patients identified from the SEER database. This model was validated, and the performance was evaluated. Calibration plots showed an optimal agreement between the observed risks and the estimated risks by the nomogram, indicating the reliability of this model. Discrimination was evaluated by the C-index and AUC values. Both of them indicated good specificity and sensitivity in this nomogram.

Several nomograms have been developed to predict the survival of MBC patients [17–20]. C. K. Lee et al. focused on predicting survival of MBC patients with relapsed disease [17]; Giovanni Corso et al. used a nomogram to predict the risk of developing relapsed disease [20]; studies by S. R. Li et al. and Z. C. Xiong et al. combined the de novo MBC patients and those with relapsed disease [18, 19]. However, many studies have shown that women with de novo MBC represent a group that is distinct from that of women with relapsed breast cancer [21–23] . Patients with de novo MBC usually have better survival than those developed from regional diseases. One hypothesis explaining the better outcome of de novo MBC than recurrent MBC is the use of adjuvant systemic therapy in patients with relapsed disease. Due to the selection of more resistant or aggressive clones during adjuvant therapy, the metastatic disease of recurrent MBC becomes more resistant to therapy. Thus, recurrent MBC patients should not be mixed together with de novo MBC patients. In our study, we only included de novo MBC patients; to our knowledge, it was the first nomogram to predict survival of patients with de novo MBC.

MBC is a kind of heterogeneous diseases. Many factors affect the prognosis and therapeutic efficacy of drugs. The molecular subtype is a vital prognostic factor and serves as the cornerstone of treatment [6, 24, 25]. According to the expression condition of ER, PR and HER2, breast cancer can be divided into four subtypes—HR+/HER2-, HR−/HER2+, HR+/HER2+ and TNBC characterized by the absence of ER, PR and HER2. In our analysis, HR+/HER2- was the most common (57.6%) subtype among MBC patients, followed by HR+/HER2+ (19.0%) and TNBC (13.6%) while HR−/HER2+ was the least common (9.8%) subtype. In the nomogram, molecular subtype played a major role in the scoring system. TNBC subtype yielded the highest score, consistent with previous reports [5, 24, 26]. The site of distant metastasis was reported to be correlated with the survival of MBC patients. Patients with bone metastasis showed the best prognosis and those with brain metastasis showed the worst prognosis [5, 27–29]. The score distribution of metastasis in the nomogram showed consistent results. It also has been reported that among MBC patients, molecular subtype correlated tightly with the preferred metastatic site [5, 27, 30]. Even in patients metastatic to the same site, molecular subtype showed a significant prognostic role. Age at diagnosis, marital status and differentiation grade also had an impact on survival. In our analysis, we combined all these prognostic factors to construct the nomogram, in order to predict the survival of a specific patient with de novo MBC accurately and identify patients with favorable prognosis. Those at a low risk of mortality should be given aggressive multidisciplinary therapy.

MBC is considered incurable. Systemic therapy remains the mainstay of therapy [7]. Over the past 2 decades, survival of MBC patients has improved dramatically due to the development of target therapy and palliative care [31–33]. In our analysis, the 1-, 3-, and 5-year OS rates were 74.5, 45.3, and 28.2%, respectively. However, the prognostic role of primary tumor resection has not been determined. In this study, we found MBC patients benefited from surgery of the primary tumor. This finding was in agreement with conclusions reported in other retrospective studies [9, 34, 35]. Due to the selection bias existing in retrospective studies, the protective role of surgery couldn’t be directly concluded. Prospective randomized clinical trials have investigated the role of primary tumor resection in MBC patients, and resulted in contradictory conclusions [36, 37]. These results indicated that primary tumor resection did improve the survival of a subset of patients, but we have to determine who should receive primary tumor resection and when to administer the surgery.

There existed some limitations in this study. Firstly, it was a retrospective study and it was subject to all the inherent biases associated with this type of study design. Furthermore, some prognostic factors were not included in the SEER database, including the number of metastatic lesions, use of endocrine therapy and use of target therapy. Thirdly, the nomogram in our study was validated in the same population and such validation on model performance could be biased. Therefore, the predictive effect of the nomogram needs to be assessed carefully in other cohorts.

## Conclusion

The developed nomogram reliably predicted OS in patients with de novo MBC and presented a favorable discrimination ability. Using this model, the role of primary tumor surgery and other significant prognostic factors in MBC patients could be estimated. This will guide surgical decision making in clinical practice, although the findings require additional validation.

## Data Availability

These data were publicly available for use in accordance with a limited use agreement for SEER research data: Surveillance, Epidemiology, and End Results (SEER) Program (https://seer.cancer.gov) SEER*Stat Database.

## Abbreviations

MBC: Metastatic breast cancer
OS: Overall survival
SEER: Surveillance, Epidemiology, and End Results
AIC: Akaike Information Criterion
AUC: Area under the time-dependent receiver operating characteristic curve
AJCC: Derived American Joint Committee on Cancer
IDC, ILC: Infiltrating duct or/and lobular carcinoma
BCS: Breast conservation surgery
ER: Estrogen receptor
PR: Progesterone receptor
HR: Hormone receptor
HER2: Human epidermal growth factor receptor-2
TNBC: Triple-negative breast cancer

## References

[1] Siegel RL, Miller KD, Jemal A. Cancer statistics, 2018. 2018; 68(1): 7–30. 10.3322/caac.21442 29313949

[2] Ferlay J, Soerjomataram I, Dikshit R, Eser S, Mathers C, Rebelo M (2015). Cancer incidence and mortality worldwide: sources, methods and major patterns in GLOBOCAN 2012. Int J Cancer.

[3] Ravdin PM, Siminoff LA, Davis GJ, Mercer MB, Hewlett J, Gerson N (2001). Computer program to assist in making decisions about adjuvant therapy for women with early breast cancer. J Clin Oncol.

[4] Paik S, Shak S, Tang G, Kim C, Baker J, Cronin M (2004). A multigene assay to predict recurrence of tamoxifen-treated, node-negative breast cancer. N Engl J Med.

[5] Gong Y, Liu YR, Ji P, Hu X, Shao ZM (2017). Impact of molecular subtypes on metastatic breast cancer patients: a SEER population-based study. Sci Rep.

[6] Lobbezoo DJ, van Kampen RJ, Voogd AC, Dercksen MW, van den Berkmortel F, Smilde TJ (2013). Prognosis of metastatic breast cancer subtypes: the hormone receptor/HER2-positive subtype is associated with the most favorable outcome. Breast Cancer Res Treat.

[7] Bernard-Marty C, Cardoso F, Piccart MJ (2004). Facts and controversies in systemic treatment of metastatic breast cancer. Oncologist.

[8] Falkson G, Holcroft C, Gelman RS, Tormey DC, Wolter JM, Cummings FJ (1995). Ten-year follow-up study of premenopausal women with metastatic breast cancer: an eastern cooperative oncology group study. J Clin Oncol.

[9] Babiera GV, Rao R, Feng L, Meric-Bernstam F, Kuerer HM, Singletary SE (2006). Effect of primary tumor extirpation in breast cancer patients who present with stage IV disease and an intact primary tumor. Ann Surg Oncol.

[10] Rapiti E, Verkooijen HM, Vlastos G, Fioretta G, Neyroud-Caspar I, Sappino AP (2006). Complete excision of primary breast tumor improves survival of patients with metastatic breast cancer at diagnosis. J Clin Oncol.

[11] Blanchard DK, Shetty PB, Hilsenbeck SG, Elledge RM (2008). Association of surgery with improved survival in stage IV breast cancer patients. Ann Surg.

[12] Shien T, Kinoshita T, Shimizu C, Hojo T, Taira N, Doihara H (2009). Primary tumor resection improves the survival of younger patients with metastatic breast cancer. Oncol Rep.

[13] Iasonos A, Schrag D, Raj GV, Panageas KS (2008). How to build and interpret a nomogram for cancer prognosis. J Clin Oncol.

[14] Balachandran VP, Gonen M, Smith JJ, DeMatteo RP (2015). Nomograms in oncology: more than meets the eye. Lancet Oncol.

[15] Posada D, Buckley TR (2004). Model selection and model averaging in phylogenetics: advantages of akaike information criterion and bayesian approaches over likelihood ratio tests. Syst Biol.

[16] Wagenmakers EJ, Farrell S (2004). AIC model selection using Akaike weights. Psychon Bull Rev.

[17] Lee CK, Hudson M, Stockler M, Coates AS, Ackland S, Gebski V (2011). A nomogram to predict survival time in women starting first-line chemotherapy for advanced breast cancer. Breast Cancer Res Treat.

[18] Li S, Zhao J, Zhu L, Su F, Chen K (2017). Development and validation of a nomogram predicting the overall survival of stage IV breast cancer patients. Cancer Med..

[19] Xiong Z, Deng G, Huang X, Li X, Xie X, Wang J (2018). Score for the survival probability in metastasis breast Cancer: a Nomogram-based risk assessment model. Cancer Res Treat.

[20] Corso G, Maisonneuve P, Massari G, Invento A, Pravettoni G, De Scalzi A (2020). Validation of a novel Nomogram for prediction of local relapse after surgery for invasive breast carcinoma. Ann Surg Oncol.

[21] Lobbezoo DJ, van Kampen RJ, Voogd AC, Dercksen MW, van den Berkmortel F, Smilde TJ (2015). Prognosis of metastatic breast cancer: are there differences between patients with de novo and recurrent metastatic breast cancer?. Br J Cancer.

[22] Dawood S, Broglio K, Ensor J, Hortobagyi GN, Giordano SH (2010). Survival differences among women with de novo stage IV and relapsed breast cancer. Ann Oncol.

[23] Yardley DA, Kaufman PA, Brufsky A, Yood MU, Rugo H, Mayer M (2014). Treatment patterns and clinical outcomes for patients with de novo versus recurrent HER2-positive metastatic breast cancer. Breast Cancer Res Treat.

[24] Carey LA, Perou CM, Livasy CA, Dressler LG, Cowan D, Conway K (2006). Race, breast cancer subtypes, and survival in the Carolina breast Cancer study. JAMA.

[25] Kennecke H, Yerushalmi R, Woods R, Cheang MC, Voduc D, Speers CH (2010). Metastatic behavior of breast cancer subtypes. J Clin Oncol.

[26] Lin NU, Vanderplas A, Hughes ME, Theriault RL, Edge SB, Wong YN (2012). Clinicopathologic features, patterns of recurrence, and survival among women with triple-negative breast cancer in the national comprehensive Cancer network. Cancer..

[27] Kast K, Link T, Friedrich K, Petzold A, Niedostatek A, Schoffer O (2015). Impact of breast cancer subtypes and patterns of metastasis on outcome. Breast Cancer Res Treat.

[28] Ording AG, Heide-Jorgensen U, Christiansen CF, Norgaard M, Acquavella J, Sorensen HT (2017). Site of metastasis and breast cancer mortality: a Danish nationwide registry-based cohort study. Clin Exp Metastasis..

[29] Lee ES, Jung SY, Kim JY, Kim JJ, Yoo TK, Kim YG (2016). Identifying the potential long-term survivors among breast cancer patients with distant metastasis. Annals Oncol.

[30] Wu Q, Li J, Zhu S, Wu J, Chen C, Liu Q (2017). Breast cancer subtypes predict the preferential site of distant metastases: a SEER based study. Oncotarget..

[31] O'Leary B, Finn RS, Turner NC (2016). Treating cancer with selective CDK4/6 inhibitors. Nat Rev Clin Oncol.

[32] Ma F, Ouyang Q, Li W, Jiang Z, Tong Z, Liu Y, et al. Pyrotinib or Lapatinib Combined With Capecitabine in HER2-Positive Metastatic Breast Cancer With Prior Taxanes, Anthracyclines, and/or Trastuzumab: A Randomized, Phase II Study. J Clin Oncol. 2019:Jco1900108. 10.1200/JCO.19.00108 31430226

[33] Andre F, Slimane K, Bachelot T, Dunant A, Namer M, Barrelier A (2004). Breast cancer with synchronous metastases: trends in survival during a 14-year period. J Clin Oncol.

[34] Hazard HW, Gorla SR, Scholtens D, Kiel K, Gradishar WJ, Khan SA (2008). Surgical resection of the primary tumor, chest wall control, and survival in women with metastatic breast cancer. Cancer..

[35] Bafford AC, Burstein HJ, Barkley CR, Smith BL, Lipsitz S, Iglehart JD (2009). Breast surgery in stage IV breast cancer: impact of staging and patient selection on overall survival. Breast Cancer Res Treat.

[36] Badwe R, Hawaldar R, Nair N, Kaushik R, Parmar V, Siddique S (2015). Locoregional treatment versus no treatment of the primary tumour in metastatic breast cancer: an open-label randomised controlled trial. Lancet Oncol.

[37] Soran A, Ozmen V, Ozbas S, Karanlik H, Muslumanoglu M, Igci A (2016). A randomized controlled trial evaluating resection of the primary breast tumor in women presenting with de novo stage IV breast cancer: Turkish Study (Protocol MF07–01). J Clin Oncol.

